# Evaluation of Mycotoxin Screening Tests in a Verification Study Involving First Time Users

**DOI:** 10.3390/toxins11020129

**Published:** 2019-02-20

**Authors:** Veronica M. T. Lattanzio, Christoph von Holst, Vincenzo Lippolis, Annalisa De Girolamo, Antonio F. Logrieco, Hans G. J. Mol, Michelangelo Pascale

**Affiliations:** 1 Institute of Sciences of Food Production, National Research Council of Italy, Via Amendola, 122/O, 70126 Bari, Italy; vincenzo.lippolis@ispa.cnr.it (V.L.); annalisa.degirolamo@ispa.cnr.it (A.D.G.); antonio.logrieco@ispa.cnr.it (A.F.L.); 2 European Commission, Joint Research Centre (JRC), Retieseweg 111, 2440 Geel, Belgium; Christoph.VON-HOLST@ec.europa.eu; 3 RIKILT-Wageningen University and Research, Akkermaalsbos 2, 6708 WB Wageningen, The Netherlands; hans.mol@wur.nl

**Keywords:** mycotoxins, screening, validation, immunoassay, mass spectrometry, cereals

## Abstract

Rapid screening methods are currently recognized as a strategic tool for mycotoxin issues management. Specific guidelines for validation and verification of mycotoxin screening methods are set in the Commission Regulation (EU) No 2014/519. This regulation establishes that the “aim of the validation is to demonstrate the fitness-for-purpose of the screening method” and focuses the entire validation procedure on determining specific cut-off values ensuring a maximum rate of false negative results of 5%. In addition, the assessment of the rate of false suspect results is addressed. With regard to rapid test-kits, ‘fitness-for-purpose’ includes not only the criteria more commonly considered when discussing laboratory-based methods (specificity, accuracy, and precision), but also more “practical” parameters such as speed and ease of implementation in a new operational environment. The latter means demonstrating under local conditions that performance parameters, as established during the validation, can be achieved by first time users. This goal can be achieved through “method verification”. The aim of the present study was to verify the fitness-for-purpose of mycotoxin screening methods when applied by first time users. This was achieved in one laboratory facility via results of a training course with multiple technicians attending. The verification study was organized similarly to a collaborative exercise and involved two groups comprising of 10 technicians each that used the methods for the first time. Different screening methods were applied for deoxynivalenol (DON) in wheat, which was mainly Enzyme Linked Immunosorbent Assay (ELISA), lateral flow device (LFD), fluorescence polarization immunoassay (FPIA), and liquid chromatography-high resolution mass spectrometry (LC-HRMS). An additional verification was done for aflatoxin B_1_ (AFB_1_) in maize and wheat using LFD and LC-HRMS, respectively. The results of analyses were used to calculate intermediate precision (RSD_ip_, covering the inter-analyst variability in preparing the analytical samples and the precision under repeatability conditions) cut-off values and false suspect rates. RSD_ip_ ranged from 6.5% to 30% for DON, and from 16% to 33% for AFB_1_. The highest obtained variances were associated with the AFB_1_ analyses due to working with much lower mass fractions. The rate of false suspect results were lower than 0.1% for all tested methods. All methods showed a fit-for-purpose method performance profile, which allowed a clear distinction of samples containing the analytes at the screening target concentration (STC) from negative control samples. Moreover, the first time users obtained method performances similar to those obtained for validation studies previously performed on the screening methods included in the training course.

## 1. Introduction

Rapid methods are routinely used in the screening of commodities for mycotoxins and they are currently recognized as a strategic tool for food companies to validate and/or verify the efficacy of their food safety management systems [1]. Screening methods are laid down in the Commission Decision (EC) No 2002/657 as ‘methods that are used to detect the presence of a substance or class of substances at the level of interest’ [2]. These methods have the capability for a high sample throughput screening of samples for potential non-compliant results. Following a classification as non-compliant (suspect), samples are subjected to confirmatory analysis while negative (compliant) samples are no further analyzed and considered compliant.

The most widely investigated rapid screening approaches for mycotoxin detection are antibody-based methods, which are mainly enzyme linked immune sorbent assays (ELISAs), that are commercially available for many years and typically used for raw materials [3,4,5]. In the last decade, rapid and user-friendly lateral flow devices (LFD) have appeared on the market and are becoming progressively competitive with ‘classic’ ELISA methods. As for the ELISA tests, LFDs do not require expensive instrumentation and high-level trained staff [6,7]. LFDs may include a customized reader to provide a higher sensitivity compared to the visual inspection and a quantitative assay. In some cases, LFD kits are equipped with a specific application (app) to scan the test strip with a smartphone, which further increases the test portability and on site applicability.

Fluorescence polarization immunoassay (FPIA) for mycotoxin determination is a relatively recent technique. However, several competitive assays for the major mycotoxins, primarily deoxynivalenol (DON), have been reported in the literature during the last decade [8,9]. Furthermore, FPIA instruments are made commercially available. The principal advantages of these assays are derived from the use of a homogenous format that avoids the separation of free from antibody-bound mycotoxin, which allows for the exclusion of additional manipulation steps [9].

On the other side, liquid chromatography-mass spectrometry (LC-MS) techniques are spreading rapidly as successful solution for simultaneous screening of a large number of mycotoxins [10,11]. The suitability of LC-MS for large-scale screening offers several advantages, including the high number of analytes that may be simultaneously screened (up to hundreds [12]), and the identity confirmation of the analyte that may be achieved without repeating the analysis. Moreover, sample preparation may be minimized and, consequently, the consumption of man power facilitates high-throughput analysis. For these reasons, costs can be comparable to those of rapid test kits if they are expressed per analyte per sample. The definition of screening methods given in the Commission Decision (EC) No 2002/657 [2] emphasizes their capabilities for a high sample throughput and the avoidance of false negative results. Both characteristics can be adequately covered by LC-MS methods if properly developed and validated.

It is important to point out that a rapid test can be regarded as a reliable tool for food safety testing, provided that care is taken in the validation and subsequent verification of their use for a given mycotoxin/matrix combination. Specific guidelines for validation and verification for mycotoxin screening methods are set in the Commission Regulation (EU) No 2014/519 [13]. This regulation establishes that the “aim of the validation is to demonstrate the fitness-for-purpose of the screening method” and focuses the entire validation procedure on determining specific cut-off values, which ensures a maximum rate of false negative results of 5% and the assessment of the rate of false suspect results. Performance characteristics such as sensitivity, selectivity, trueness, and precision are embedded in these parameters, since cut-off values and the distance of these values from samples with mycotoxins mass fractions far below the legal limits directly depend on these characteristics.

With regard to rapid test kits, ‘fitness-for-purpose’ includes not only the criteria more commonly considered when discussing laboratory-based methods (limit of detection, specificity, trueness, and precision), but also more “practical” parameters such as speed and ease of implementation in a new operational environment. While the above mentioned performance characteristics are of particular importance when comparing results from rapid test kits and analyses undertaken by official and/or accredited procedures, speed or sample throughput are important criteria to be considered from the point of view of food business operations. Furthermore, a rapid method that fits the capability of the user has more chance of being selected for implementation. In particular, this means demonstrating under local conditions that first time (non-experienced) users can achieve performance parameters established during the validation. This goal can be achieved through “method verification”. During method verification, the laboratory (technician) is required to demonstrate that it can achieve certain performance parameters established during the validation study [14]. For screening methods, critical requirements to be verified are the cut-off values and rate of false suspect and false negative results. Verification of a laboratory/technician ability to properly carry out a screening method can be demonstrated by analyzing negative and positive (incurred or fortified) samples [13].

The Commission Regulation (EU) No 519/2014 foresees a specific experimental design for verification to be applied by single laboratories that intend to use a screening test that has been previously subjected to an inter-laboratory validation exercise. As a minimum sample set, the regulation suggests to include six negative control samples (i.e., known to be free of the mycotoxin of interest) and six control samples contaminated at levels above the screening target concentration (STC), typically the EU maximum level (ML), if available. Results comparable to those reported for the validated method demonstrate the laboratory’s ability to correctly apply the method. More specifically, when analyzing the positive STC control samples, the results of all samples must be above the cut-off value specified in the validation report, which correctly classifies these samples as suspect positive. When this criterion is not met, the laboratory must perform a root-cause analysis to identify why it could not meet the specification obtained in the inter-laboratory trial and take corrective actions. Moreover, the verification guideline in this regulation foresees that the laboratory can perform a validation exercise if a comparison with results from a collaborative trial is not possible.

The aim of the present study was to verify the fitness-for-purpose of different mycotoxin screening methods. This was done in one laboratory facility with multiple technicians attending a one-week workshop-training course on rapid methods for mycotoxin detection in the food chain. The training course was held at the Institute of Sciences of Food Production, National Research Council of Italy, in October 2017. The verification study was organized similarly to a collaborative exercise and involved two groups comprised of 10 technicians each that used the methods for the first time. Four different screening methods were applied including FPIA, ELISA, LFD, and liquid chromatography-high resolution mass spectrometry (LC-HRMS) for DON screening in wheat. In addition, LFD and LC-HRMS were also applied for aflatoxin B_1_ (AFB_1_) determination in maize and wheat, respectively. The results of analysis were statistically evaluated to calculate precision, cut-off values and the rate of false suspect results. The obtained performance results were interpreted in terms of correct classification of the samples as negative and suspect-positive ones. Results of the statistical evaluation were then compared with those obtained from previous validation studies carried out using the same screening methods applied in the present study. Lastly, the statistical analysis of the results (ANOVA) allowed us to draw some consideration of major factors affecting the method precision. This evaluation was carried out on data obtained from the analysis of the STC positive samples since only these results are linked to the measurement of the target analytes, in contrast to the analysis of negative samples.

## 2. Results

When evaluating different screening methods, the rates of false negative and suspect results along with the calculated cut-off values are the key parameters to consider. In addition, the precision and the recovery rates obtained in the training were included in the assessment of the methods since these characteristics have a direct impact on the cut-off values. For instance, when the values for the precision are low and the recovery rates are high, the cut-off values are close to the STC, which ensures low rates for the false suspect results of compliant samples.

**Figure 1:**
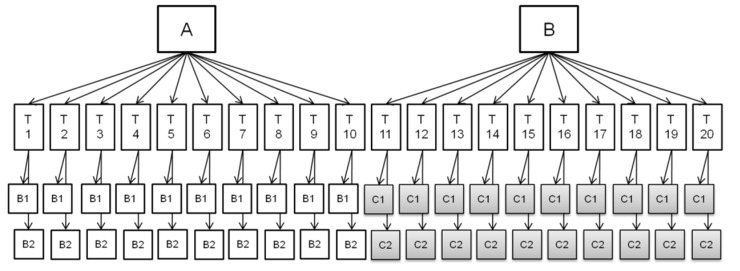
Scheme of the experimental design applied in the verification exercise. A, B: groups (10 technicians per each group); T: technician. B1, B2: negative samples (two replicates analyzed by each technician). C1, C2: samples contaminated at STC (two replicates analyzed by each technician).

**Figure 2:**
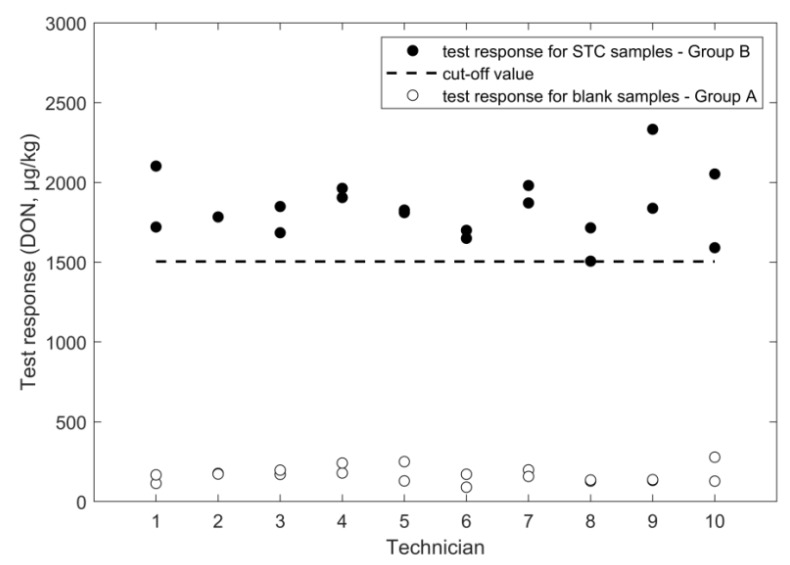
FPIA results for DON analysis in wheat negative and STC (1600 µg/kg) samples.

**Figure 3:**
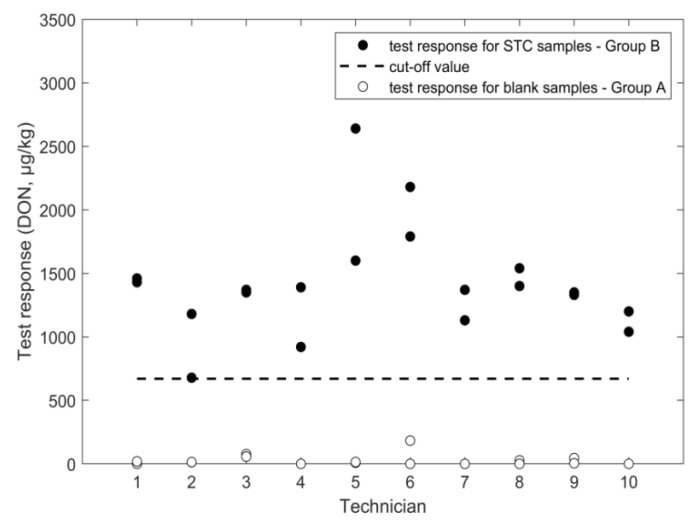
ELISA results for DON analysis in wheat negative and STC (1600 µg/kg) samples.

**Figure 4:**
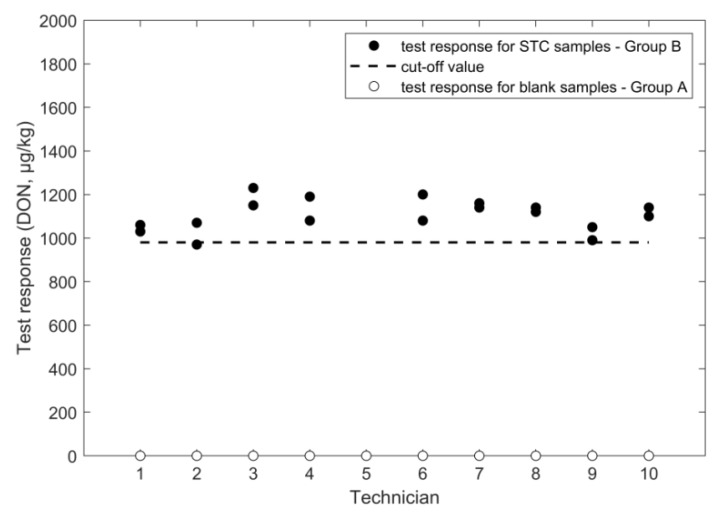
LFD results for DON analysis in wheat negative and STC (1600 µg/kg) samples.

**Figure 5:**
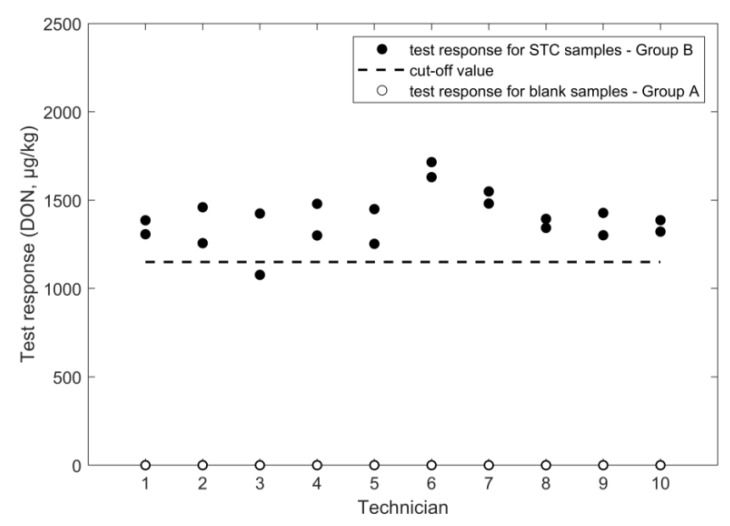
LC-HRMS results for DON analysis in wheat negative and STC (1600 µg/kg) samples.

**Figure 6:**
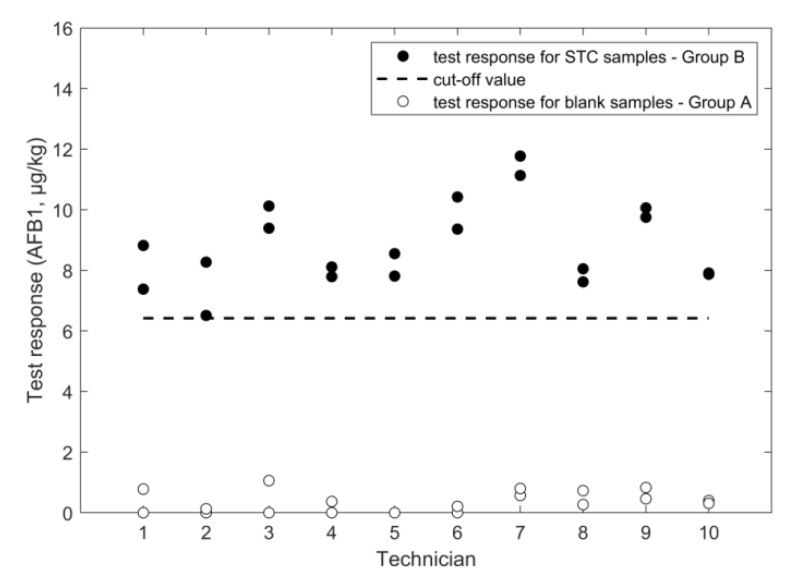
LFD results for AFB_1_ analysis in maize negative and STC (10.6 µg/kg) samples.

**Figure 7:**
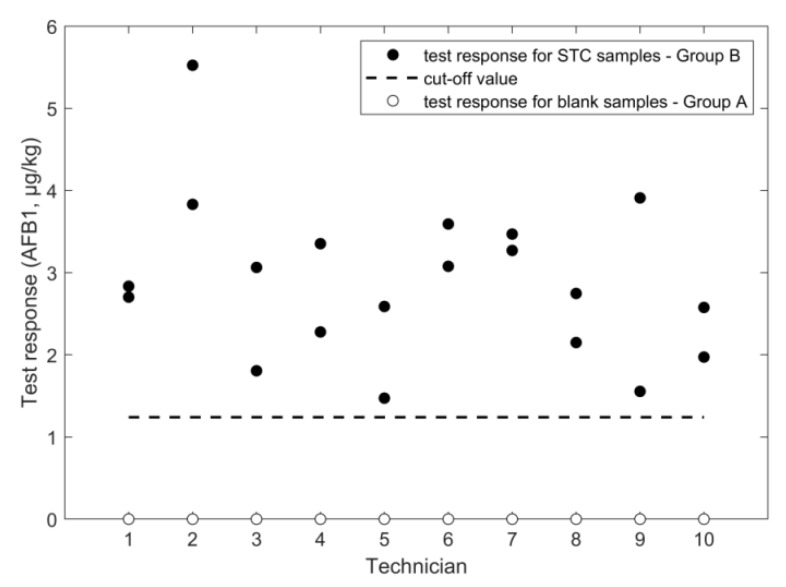
LC-HRMS results for AFB_1_ analysis in wheat negative and STC (2 µg/kg) samples.

An overview of the outcome of the study, performed according to the experimental design in Figure 1, is given in Figure 2, Figure 3, Figure 4, Figure 5, Figure 6 and Figure 7, in which the analytical results obtained by the technicians for the duplicate negative samples and the duplicate contaminated samples are shown. The figures also indicate the cut-off value calculated for each method after statistical assessment of the analytical results of the STC samples by applying Equation (3). The visual inspection of the figures confirmed that, in all cases, the negative samples and the STC samples formed two groups that were clearly separated by the calculated cut-off values. For all methods, there were some results close to the cut-off value or for even a single analytical result of the STC samples wrongly below the cut-off values (Figure 2, Figure 3, Figure 4, Figure 5, Figure 6 and Figure 7). However, given the total number of 20 data, such a result can be expected since an acceptable rate of false negative results of 5% was selected, which means that one out these 20 values may be below the cut-off value. Furthermore, for all methods, none of the results of the negative samples was above the cut-off value. Therefore, no false suspect results were observed.

The results from the statistical assessment and a comparison with corresponding values from previous studies are summarized in Table 1 and Table 2, respectively. The reported results of the technicians of the training course were subjected to ANOVA without any removal of potential outliers. In the following paragraphs, the results from the statistical assessment of the data are separately discussed for each method.

### 2.1. Determination of Deoxynivalenol

#### 2.1.1. FPIA

The values for the relative standard deviation for repeatability (RSD_r_) and for intermediate precision (RSD_ip_) calculated from the results of the STC samples were 10% in both bases (Table 1). The obtained precision is considered acceptable even though they were higher compared to the RSD_r_ of 4.1% gained in the single laboratory validation (Table 2) [15]. In addition, the obtained recovery of 115% was considered acceptable and comparable with a value of 98% that was reported from the previously conducted single-laboratory validation (Table 2) [15]. Due to the very good performance of the method in the training course, the calculated cut-off values was 1504 µg/kg that is close to the STC (1600 µg/kg) (Figure 2). From the results of the negative samples, the rate of false suspect results was estimated to be below 0.1% (Table 1).

#### 2.1.2. ELISA

While the study revealed an acceptable value for the mean response at STC (recovery rate of 89%) and a repeatability of 21% (similar to 22% repeatability obtained in previous validation), the RSD_ip_ was 30% and, therefore, higher compared to the other methods (Table 1). Moreover, the statistical assessment from ANOVA revealed that the contribution of the different technicians to the overall variance of the results was about 63% (data not shown) and, therefore, higher than the contribution of the variance obtained when the method was performed by the same technician. This may hint at the specific format of the ELISA method, which requires the precise execution of many more steps compared to LFD or FPIA. In consequence, small deviations from the protocol when applied by different technicians can lead to a higher scattering of the analytical results. By taking into account Equation (3), the higher variation of the results led to a low cut-off value, which was 674 µg/kg (Table 1). However, this value was still high enough to gain a clear separation from the result of the blank samples (Figure 3). The rate of false suspect results calculated from the results of the negative samples was below 0.1%.

#### 2.1.3. LFD

Very good values for the precision were obtained in the training course since the RSD_r_ was 4.8% and the RSD_ip_ was 6.5% (Table 1), which was even lower than the corresponding value of 13% from the previous validation study (Table 2). On the other hand, the mean value calculated from the results from STC samples in the study was low, as indicated by a recovery rate of 69%. Moreover, this low mean value led to a cut-off value of 980 µg/kg, which was below the cut-off value of 1410 µg/kg obtained in the previous validation study (Table 2). As recommended in the Commission Regulation (EU) No 2014/519, a root cause analysis should be performed when performance characteristics obtained in verification experiments do not coincide with specifications established in previous validation studies. The evaluation indicated that one reason for the drop of the recovery rate could be a deviation from the protocol in the training course because, for practical considerations, samples were extracted by manual shaking, which is supposed to be less efficient when compared to actual foreseen mechanical extraction using a vortex. It is, therefore, recommended that vortex extraction should be applied when implementing the method under routine conditions. The analysis of the negative samples did not deliver a numerical value and, therefore, the rate of false suspect results could not be calculated. Similar analytical performances were obtained in a previous study for DON determination in wheat by lateral flow manufactured by another company [19], which indicates the robustness of the currently well-established LFD technology from different brands.

#### 2.1.4. LC-HRMS

The statistical assessment of the technicians’ results delivered a good method performance profile, since the RSD_r_ was 8.4% and the RSD_ip_ was 10% (Table 1), while the recovery rate was 87%. The method has been previously evaluated by an interlaboratory study organized in the framework of the EC standardization mandate M520/EN for methods of analysis for mycotoxins in food (prEN 17279:2018 Foodstuffs—Multimethod for the screening of aflatoxin B1, deoxynivalenol, fumonisin B1 and B2, ochratoxin A, HT-2, and T-2 toxins and zearalenone in foodstuffs, excluding foods for infants and young children, by HPLC-MS/MS) [17], which obtains similar values of 3.4% for the RSD_r_ and of 11% for the RSD for reproducibility (RSD_R_) (Table 2). Given the acceptable values for the precision and the recovery rate in the training course, the cut-off values was 1151 µg/kg, which was close to the STC of 1600 µg/kg of the training course (Figure 5). Since the STC of 250 µg/kg chosen in the previous study was much lower, a comparison of the results in both studies may be possible only considering the relative cut-off values, i.e., calculated by dividing the obtained cut-off value by the STC considered in the study. The relative cut-off values obtained in the inter-laboratory study and in the training course were 0.74 and 0.72, respectively, which indicates very similar method performances in the two tested ranges.

### 2.2. Determination of Aflatoxin B_1_

#### 2.2.1. LFD

The statistical assessment of the data from the training course delivered acceptable values for the RSD_r_ and RSD_ip_, which were 7.2% and 16%, respectively. Based on the mean response at STC, a recovery rate of 83% could be calculated. Due to 20 technicians running the samples in the same period and having only two readers set up, some student’s samples were tested at greater than five minutes of strip test development time. Variability in development time may lead to higher RSD_r_ and RSD_ip_ than expected when running under routine conditions. The RSD_ip_ of the previously performed validation study was 29% [18] and, therefore, higher compared to the training course (Table 3). However, obvious differences of the experimental design used, such as the inclusion of the between day and between matrix factors in the previous validation study, as additional error sources do not apply, since the RSDs for repeatability were quite different (Table 3). One reason could be that the STC selected in the previous study was 4 µg/kg and, therefore, lower compared to the STC of 10 µg/kg applied in the training course, which reflects the general trend that the variation of the data is increased at lower mass fractions [20]. Additionally, in this case, since the two different STC values were set in the previous study (4 µg/kg) and in the training course (10.6 µg/kg), it was possible to compare the relative cut-off values only, which were 0.55 and 0.60 (Figure 6), respectively. From the results of blank samples, the rate of false suspect was estimated to be below 0.1%.

#### 2.2.2. LC-HRMS

The variation of the measured results obtained in the training course was higher than the corresponding values from the LFD method since the RSD_r_ and RSD_ip_ were 28% and 33%, respectively (Table 1). Moreover, results from ANOVA (data not shown) showed that the influence of the application by the different technicians was minor, which indicates a source of error beyond the manual operations. The RSDs of this method are also larger compared to the corresponding values of the LC-HRMS method for DON. The main reason for this is that the signal for DON was well above the instrument LOQ whereas, for AFB_1_, it was close to the LOQ where higher RSDs (due to higher variance in areas of low intensity peaks) can be expected. The RSD_r_ observed here was higher than that observed in a previously conducted inter-laboratory study (19%, Table 2). The cut-off value obtained in the training course was 1.23 µg/kg (Figure 7) and slightly higher compared to the cut-off value of 1.08 µg/kg from the previous validation study. No false suspect results were observed on negative samples.

## 3. Discussion

### Practicability of the Screening Methods

Besides the screening performance, practicality of the screening method is also very important. Relevant aspects in this respect are the time needed for analysis, the skills or level of education of the user of the method, the place where the analysis needs to be carried out (field or laboratory environment), the variety of matrices, and the number of mycotoxins of interest. Although the latter two aspects were not investigated in detail in this paper, some comments on this are included in this section. In terms of time per sample, it should be mentioned that, before extraction, a certain amount of cereal grains needs to be ground. The amount needs to be representative for the consignment or lot. For highly heterogeneously distributed contamination like aflatoxins, this is more critical than for DON [21,22]. A sufficiently large sample size, or alternatively, more replicates of smaller sub-samples, need to be taken. LFDs are very straightforward and fast (the total analysis time including sample preparation are generally lower than 10 min), and ideal for field situations, but includes only one sample per analysis/strip. If LFDs are intended for in field deployment, and, therefore, under variable conditions, the main environmental parameters potentially affecting the reliability of the assay (temperature, matrix-to-matrix variations) should be taken into account at the validation (or verification) stage. The analysis of reference materials alongside any samples may help keep the analytical performances under control.

FPIA is slightly less straightforward because it requires a basic laboratory environment. However, FPIA-based methods are simple, rapid, can be automated, and are suitable for high-throughput screening, as well as for reliable quantitative determination of mycotoxins. ELISAs involve more steps, require more skills, a basic laboratory environment, and more time, but can handle up to 96 samples simultaneously (calibrants included). Therefore, they are highly efficient when a large number of subsamples need to be analyzed in a short time. In LC-MS based screening, extraction can be done simultaneously for many samples. Sample analysis is sequential but automated and can be run overnight. When properly optimized, analysis times of 10 min/sample are realistic [23], which enables a throughput of >100 samples/24 h. LC-MS is also ideal for simultaneous screening of high numbers of contaminants in one sample (multiple mycotoxins/plant toxins/pesticides) and can easily handle a wide variety of matrices [24]. Lastly, even though not specifically required for screening purposes, LC-MS provides chromatographic separation and information on the molecular weight of the detected compounds, which increases confidence in analyte identification and minimizes the risk of overestimation due to aspecific response of matrix compounds. However, it requires a sophisticated laboratory environment, expensive instrumentation, and highly skilled operators.

Lastly, a possible role associated with the use of “competitive” format (LFD, ELISA, FPIA) to the assay variability and reliability should be taken into account. The overall reliability of the indirect tests (competitive immunoassays) can be affected by interferences (changes in pH, solvent, and, in general, environmental parameters) or small deviations in the analysis protocol, such as variations in the time associated with incubation steps, needed to approach the antigen/antibody equilibrium. Direct tests, such as mass spectrometric determinations, are less subjected to the factors mentioned above. However, they imply extra costs and technician training to ensure proper instrument management, calibration and maintenance, and correct data processing. As a general consideration about analysis costs, it is realistic to estimate the costs of immunoassay analysis to be, on average, 10 times lower than those related to LC-MS.

From the above considerations, it is clear that the most suitable screening method depends on the situation and its intended purpose.

## 4. Conclusions

In the current study, six different methods for determining specific mycotoxins have been successfully evaluated within a training course, which covers four different analytical formats known as FPIA, ELISA, LFD, and LC-HRMS. All methods showed a fit-for-purpose method performance profile, which allowed a clear distinction of samples containing the analytes at the STC from negative control samples. In addition, the first time users obtained overall method performances similar to those obtained during validation studies. While there are differences in terms of the observed precision, the fitness-for-purpose of the individual methods depends on additional factors such as the circumstances under which the methods are applied. For instance, when looking for a method that works on site, i.e., without a laboratory and high skilled personnel at hand, LFD is the first choice. On the other hand, when implementing a screening method in a well-equipped laboratory, ELISA, FPIA, and LC-HRMS show some striking advantages in terms of high sample throughput for the formers and automation for the latter method. Moreover, it can be expected that the precision of these methods improves when running under routine conditions. Furthermore, the response of LC-HRMS has also confirmatory power, which extends the scope of the method. While the specificity of all methods has been tested on negative samples, it would be interesting to challenge the methods by analyzing samples containing the analytes below the STC, at 25% or 50%, and by including different samples from the same matrix (f.i., different wheat cultivars) or different types of matrices (e.g., different cereals and processed cereals). The selection of such “sub” STC levels, however, should take into account typical levels of the mycotoxins in the target matrices. Given the increasing role of screening methods in the control of food, the organization of proficiency tests specifically designed to this category of methods should be considered.

## 5. Materials and Methods

### 5.1. Test Materials

According to the experimental design applied in the training course outlined in Section 5.3, each technician had to analyze blind duplicates of one negative or one contaminated sample, for each of the tested methods (Figure 1). The mass fraction of the target mycotoxin in the contaminated samples was in the range of the EU maximum limit (ML) for the matrix analyzed. All samples were milled (<0.5 mm).

For FPIA, ELISA, LFD, and LC-HRMS determination of DON and AFB_1_ in wheat:

(a) Uncontaminated (negative) wheat (DON ≤ 100 µg/kg, AFB_1_, AFB_2_, AFG_1_, AFG_2_ ≤ 0.5 µg/kg), reference material from Trilogy^®^, batch # D-W-100

(b) Wheat contaminated with 1600 ±300 µg/kg DON (i.e., close to the ML for DON in durum wheat, 1750 µg/kg) reference material from Trilogy^®^, batch #D-W-177

For the LC-HRMS method targeting AFB_1_, contaminated wheat was obtained by the spiking test material (b) at the mass fraction of 2 µg/kg of AFB_1_ (i.e., the ML for AFB_1_ in wheat)

For LFD determination of AFB_1_ in maize:

(c) Uncontaminated (negative) maize (AFB_1_, AFB_2_, AFG_1_, AFG_2_ ≤ 0.5 µg/kg) obtained from the Italian retail market

(d) Maize contaminated with 10.6 ± 2.0 µg/kg total aflatoxins (i.e., the ML for total aflatoxins in maize to be subjected to sorting), reference material from Trilogy^®^, batch # A-C-2211

The AOAC Official Method 991.31 assessed the absence of aflatoxins in test materials a) and c) with a limit of quantification of 0.5 µg/kg for AFB_1_, AFB_2_, AFG_1_, and AFG_2_ individually.

### 5.2. Description of the Analytical Methods

The protocols of each screening method applied are described in the following paragraphs. For comparison purposes, Table 3 reports the main steps of each procedure.

#### 5.2.1. FPIA—Deoxynivalenol in Wheat

Samples were analyzed, according to the procedures described by Lippolis et al. and Valenzano et al. [15,25].

Sample preparation. Twenty-five g of test material were weighed into a 250 mL blender jar and extracted with 100 mL PBS (phosphate buffered saline, from Sigma-Aldrich, Milan, Italy) solution by blending at high speed for 2 min. The extract was filtered through both paper filter and glass microfiber filter.

FP immunoassay. A volume of 830 µL of PBS solution was pipetted into a test tube, followed by 80 µL of DON-antibody (monoclonal antibody clone 22 by the U.S. Department of Agriculture–Agricultural Research Service–National Center for Agricultural Utilization Research, Peoria, Ill.) working solution and 120 µL of filtered extract and the mixture was vortexed. The tube was placed into the FP reader to read the blank. Then 25 µL of tracer solution (DON labeled with fluorescein, DON-FL) were added. After 2 min of incubation, the tube was placed into the FP reader. The FP reader calculated and displayed the polarization value expressed in milli-polarization units.

Determination of DON mass fraction. The calibration curve was prepared by the training course organizers. Each technician read the polarization value of the test sample and entered this value in an excel sheet calculating the DON mass fraction.

Equipment. The instrument to measure the fluorescence polarization was the Sentry FP 100 (Diachemix LLC) equipped with filters (excitation wavelength 485 nm and emission wavelength 535 nm). Polarization measurements were performed after incubation (2 min).

#### 5.2.2. ELISA—Deoxynivalenol in Wheat

Samples were analyzed, according to the procedures provided by the manufacturer-biopharm AG (Darmstadt, Germany). Calibration standards, conjugate, antibody, and substrate/chromogen solutions were provided in the kit.

Sample preparation. Five grams of the test sample were weighed into a tube and extracted with 100 mL of distilled water by manual shaking for 3 min. The extract was filtered through filter paper.

ELISA. A total of 50 µL of calibration standards or the test sample extract were pipetted into separate wells. Then, 50 µL of conjugate solution were added to each well, which was followed by 50 µL of antibody solution. The plate was gently shaken manually and incubated for 5 min at room temperature. After discarding the liquid by turning it upside down, the wells were washed three times with 250 µL of washing buffer. An aliquot of 100 µL of substrate/chromogen solution were added to each well, and the plate was gently shaken manually to mix. After 3 min of incubation at room temperature in the dark, 100 µL of stop solution were added to each well.

Determination of DON mass fraction. Each technician prepared his own calibration curve. For each well (calibration standards and test sample), the absorbance at 450 nm was measured by a plate reader. The DON mass fraction was obtained by inserting the absorbance values in the software provided by the supplier.

Equipment. The ELISA kit (RIDASCREEN^®^FAST DON) was from r-biopharm AG (Darmstadt, Germany), the Multiskan MS Plus MK II ELISA reader from Labsystems (Helsinki, Finland).

#### 5.2.3. LFD—Deoxynivalenol in Wheat

Samples were analyzed according to the procedures provided by the manufacturer r-biopharm AG (Darmstadt, Germany).

Sample preparation. One gram of the test sample was weighed into a screw cap tube and extracted with 40 mL of extraction buffer (provided in the kit) by manually shaking for 3 min. The extract was allowed to settle for 3 to 5 min and then filtered through filter paper.

LFD analysis. An aliquot of 100 µL of the clear supernatant was applied onto the application area of the test strip and allowed to develop for 5 min.

Determination of DON mass fraction. Intensities of the test line and control line developed on the strip membrane were measured using a reading system made by a smartphone and a specific software provided by the supplier. The ratio of the test and control lines was converted into DON mass fraction through a lot-specific calibration curve uploaded onto the smartphone system by using the corresponding barcode provided by the supplier.

Equipment. The immuno-chromatographic test (RIDA^®^QUICK DON) and the smartphone software (RIDA^®^SMART APP) were from r-biopharm AG (Darmstadt, Germany).

#### 5.2.4. LFD—Aflatoxin B_1_ in Maize

Samples were analyzed, according to the procedures provided by the manufacturer Vicam, A Waters Business (Milford, MA, USA).

Sample preparation. Five grams of maize were extracted with 25 mL of “AQUA Premix” solution (provided in the kit) by magnetic stirring for 3 min. The sample extract was filtered through a filter paper.

LFD analysis. An aliquot of 100 µL of filtered extract was slowly pipetted onto the strip (about one drop per second) and allowed to develop for 5 min. Next, the lateral flow device was immediately placed into the reader holder.

Determination of aflatoxin mass fraction. The intensities of the test line and control line developed on the strip membrane were measured using the photometric reader. Then, the test response was calculated as the ratio between the signal intensity of the test line and that of the control line and was converted into aflatoxin mass fraction through a lot-specific calibration curve. The lot-specific calibration curve was uploaded onto the reader system by using the corresponding barcode provided by the supplier.

Equipment. The strip test (AFLA-V AQUA™) and reader (Vertu Reader) were from VICAM (Vicam, A Waters Business, Milford, MA, USA).

#### 5.2.5. LC-HRMS—Deoxynivalenol and Aflatoxin B_1_ in Wheat

Sample preparation. Five grams of test samples were weighed into a 50 mL centrifuge tube, after which 10 mL water and 10 mL of a mixture of acetonitrile:acetic acid (99:1, *v*/*v*) were added (acetonitrile, high-performance liquid chromatography grade, and glacial acetic acid were from VWR International, Milan, Italy). The tube was closed and shaken by hand to premix sample and solvent. Then, the tubes were placed in a mechanical shaker for a 30-min extraction. After the extraction step, 5 g of magnesium sulfate (Sigma-Aldrich, Milan, Italy) was added and the tube was shaken vigorously for 30 s by hand. The tube was then centrifuged at 3000*g* for 5 min to aid the settlement of particulate matter and phase separation. The upper acetonitrile layer was used for LC-HRMS analysis. The test solution was prepared by combining into a vial 200 µL of acetonitrile extract, and 180 µL of water. Then the TC organizers added 20 µL of mixed internal standard solution (^13^C-labeled mycotoxins, Biopure Referenzen substanzen GmbH, Tulln, Austria), according to the safety rules of the TC-hosting institution.

Determination of mycotoxin mass fraction. The quantification was based on internal calibration using the response of the ^13^C-label present in each sample extract at the STC level, according to the following equation.

(1) $$C_{\mathrm{Sample}}=\frac{R_{\mathrm{Sample}}\times C_{\left(\mathrm{IS}/\mathrm{STC}\right)}}{R_{\left(\mathrm{IS}/\mathrm{STC}\right)}}$$

where:

C_Sample_ = mass fraction of the target analyte in the sample expressed in µg/kg;
R_Sample_ = response (area) of peak in extracted ion chromatogram using the exact *m*/*z* ± 5 ppm of the protonated molecule;
R_(IS/STC)_ = response (area) of peak in extracted ion chromatogram using the exact *m*/*z* ± 5 ppm of the protonated molecule of the ^13^C-label added at the STC level;
C_(IS/STC)_ = STC of ^13^C-label in the extract, expressed in the corresponding µg/kg equivalent in sample

Equipment. LC-HMRS analysis were performed on a Q-Exactive^TM^ Plus mass spectrometer, which was equipped with a heated electrospray ion source (HESI II) coupled to an Ultimate 3000 UHPLC system (all from Thermo Fisher Scientific, San Jose, CA, USA).

The LC column was an Accucore^TM^aQ (150 × 2.10 mm, 2.6 µm particles) (Thermo Fisher Scientific) preceded by an Accucore^TM^aQpre column (10 × 2.1 mm, 2.6 µm particles). The column oven was set at 40 °C. The flow rate of the mobile phase was 300 µL/min, while the injection volume was 10 µL. A gradient elution was performed by using a mixture of water (eluent A) and methanol (eluent B), which both contain 0.5% acetic acid and 1 mM ammonium acetate (Sigma-Aldrich, Milan, Italy). The HRMS analyzer operated in a full scan mode (mass range *m*/*z* 100–900 *m*/*z*, a resolving power of 70,000 FWHM, defined at 200 *m*/*z*). The system was controlled by the Xcalibur (version 3.1), and Chromeleon MS Link 6.8 and Q-Exactive Tune 2.8 software package (both from Thermo Fisher Scientific, San Jose, CA, USA, 2011).

While the technicians of the training course did the whole sample preparation procedure, a technician from the organizing laboratory operated the LC-HRMS instrument. However, by applying an automatic system for processing the chromatogram, the influence of the technician on the LC-HRMS measurement was minimized.

### 5.3. Set Up of the Validation Exercise

The purpose of the validation exercise carried out within the training course was the verification of fitness-for-purpose of mycotoxin screening methods when applied by first time users. The collaborative exercise involved 20 technicians from 11 different countries from all over the world, which represents a cross-section of research, industry, and official control laboratories that used the methods for the first time.

An overview of the experimental design applied for the verification exercise is given in Figure 1. The exercise involved two groups (group A and group B) comprising of 10 technicians each. Group A and group B had to analyze 10 negatives samples and 10 positive samples with the analyte at STC, respectively, as blind duplicates. In a total of 20 measurements for each level (i.e., 20 negative samples and 20 samples contaminated at STC) were performed.

For each method to be tested, each technician received:

1. A negative sample to be analyzed in duplicate (group A) or a sample contaminated with the target mycotoxin at STC to be analyzed in duplicate (group B)

2. Calibration standards according to the above described procedures;

3. The method protocol in the SOP format;

4. Calculation excel sheets or software to process the results, according to the above described procedures. The results depend on the specific method applied.

The work was organized within the group in such a way that each technician analyzed a pair of replicates of negative or STC positive samples. However, the technicians did not know whether they analyzed negative or positive samples. A 15-min lecture prior the practical training was given to introduce the concept of the method and to illustrate in detail all the main steps of the experimental procedure to enable the trainees to strictly follow the SOP protocol.

### 5.4. Statistics Applied

While the screening tests investigated in this study were used to classify samples into exclusively two classes, namely negative and suspect positive, these methods delivered a quantitative estimate of the mass fraction of the target analytes in the samples. Therefore, the procedure outlined in the Commission Regulation (EU) No 519/2014 was applied in which results from replicate analyses of samples containing the analytes at the STC (positive samples) are used to determine *(i)* the precision of the method, *(ii)* the overall mean value of the analytical results, and *(iii)* the cut-off value. Samples were subsequently classified as negative or suspect positive depending on whether the results of analysis were below or above the cut-off value, respectively.

Commission Regulation (EU) No 519/2014 foresees as the minimum experimental design for single-laboratory validation that 20 replicate analyses are performed on positive and negative samples, respectively. Moreover, the experiments should be carried out under intermediate precision, distributing the 20 samples over five days. The analytical results are subsequently subjected to Analysis of Variance (ANOVA) to calculate the intermediate precision of the test. This specific design could not be fulfilled within the training course, since all experiments for a specific test were carried out on the same day. However, since the different participants of the training course performed the tests, the between-technician variation could be introduced in the model underlying the ANOVA as specified here.

Y_ik_ = TV + Tech_i_ + R_ik_ (2)

where Y_ik_ is the response of the measurement, TV the true value, Tech_i_ the between technician variation, and R_ik_ is the variation under repeatability conditions.

Taking into account that, in all applied procedures, the technicians obtained the already prepared calibrants, and the between technician variation means inter-analyst variability in preparing the analytical samples.

From the results obtained on the analysis of the samples containing the analytes at the STC, the overall mean of all experiments (Mean_STC_) and the standard deviation of intermediate precision (SD_STC_) were determined. Lastly, the cut-off values were calculated, according to the following equation.

Cut-off value = Mean_STC_ − *t*-value_(0.05)_ × SD_STC_ (3)

where the *t*-value_(0.05)_ is taken from a table for a one-sided test at a significance level of 5%. This means that the cut-off value is always below the target level and, by setting the significance level at 5%, the rate of false negative results is 5% as well. According to the Regulation (EU) No 519/2014, 5% is considered to be an acceptable rate of false negative results for screening tests. Even though not specifically required by the regulation for screening methods, the obtained values for Mean_STC_ were also used to calculate the recovery rates of each method as additional information.

The purpose of the experiments conducted on the negative samples was to assess the rate of false suspect results. For those methods where the analysis of the negatives amples delivered a response rather than “<LOQ”, the obtained duplicate results were subjected to ANOVA to assess the intermediate precision (SD_neg_). From the cut-off value, the mean of the results of the analysis of the negative samples (Mean_neg_), and the intermediate precision, the *t*-value is calculated with the following equation.

(4) $$t-\text{value}=\;\frac{\left(\text{cut}-\text{off}\;\text{value}-{\mathrm{Mean}}_{\mathrm{neg}}\right)}{{\mathrm{SD}}_{\mathrm{neg}}}$$

By inserting the obtained *t*-value in the “TDIST” function of Microsoft Excel, the corresponding rate of false suspect results was calculated.

## Figures and Tables

**Table 1:** Screening method performances obtained in the training course verification study.

Sample Set	Validation Parameters	DON				AFB_1_	
		LFD Wheat	ELISA Wheat	FPIA Wheat	LC-HRMS Wheat	LFD Maize	LC-HRMS Wheat
negative samples	mean response (µg/kg)	0	23	168	n.a.	0.3	n.a.
	SD repeatability (µg/kg)	n.a.	43	52	n.a.	0.3	n.a.
	SD intermediate (µg/kg)	n.a.	43	52	n.a.	0.3	n.a.
	rate of false suspect (%)	<0.1%	<0.1%	<0.1%	<0.1%	<0.1%	<0.1%
positive samples	STC(µg/kg)	1600	1600	1600	1600	10.6	2.0
	mean response (µg/kg)	1106	1417	1833	1397	8.8	2.9
	SD repeatability (µg/kg)	53	301	186	117	0.6	0.8
	SD intermediate (µg/kg)	72	429	190	142	1.4	1.0
	RSD repeatability (%)	4.8	21	10	8.4	7.2	28
	RSD intermediate (%)	6.5	30	10	10	16	33
	cut off (µg/kg)	981	674	1504	1151	6.4	1.2

n.a. not applicable—returned value for blank “not detected” or “zero” by default.

**Table 2:** Screening method performances—comparison with previous validation studies.

Mycotoxin		Present Training Course	Previous Studies			
		Validation Parameters	Study Design	Guidelines	Validation Parameters	Reference
DON	FPIA/ wheat	STC: 1600 µg/kg intermediate precision ^TC^: 10% repeatability: 10% cut-off: 1504 µg/kg false suspects for blanks: <0.1%	single laboratory	CEN/TR 13505	STC: 1750 µg/kg recovery: 97% repeatability: 4.1% limit of detection: 80 µg/kg	Lippolis et al. [15]
	ELISA/ wheat	STC: 1600 µg/kg intermediate precision ^TC^: 30% repeatability: 21% cut-off: 670 µg/kg false suspects for blanks: <0.1%	single laboratory	AOAC performance tested	STC: 1000 µg/kg recovery: 100% repeatability: 22% limit of quantification: 167 µg/kg	r-biopharm [16]
	LFD/ wheat	STC: 1600 µg/kg intermediate precision ^TC^: 6.5% repeatability: 4.8% cut-off: 980 µg/kg false suspects for blanks: <0.1%	single laboratory	519/2014/EC	STC: 1600 µg/kg intermediate precision: 13% repeatability: 12% cut-off: 1410 µg/kg false suspects for blanks: <0.1%	unpublished results
	LC-HRMS/ wheat	STC: 1600 µg/kg intermediate precision ^TC^: 10% repeatability: 8.4% cut-off: 1151 µg/kg relative cut off: 0.71 false suspects for blanks: <0.1%	inter-laboratory	519/2014/EC	STC: 250 µg/kg reproducibility: 11% repeatability: 3.4% cut-off: 184 µg/kg *relative cut off: 0.74* false suspects for blanks: <0.1%	prEN 17279:2018 [17]
AFB_1_	LFD/ maize	STC: 10.6 µg/kg intermediate precision ^TC^: 16% repeatability: 7.2% cut-off: 6.4 µg/kg false suspects for blanks: <0.1%	single laboratory	519/2014/EC	STC: 4 µg/kg intermediate precision*: 29% repeatability: 21% cut-off: 2.18 µg/kg false suspects for blanks: 8%	Lattanzio et al. [18]
	LC-HRMS/ wheat	STC: 2 µg/kg intermediate precision ^TC^: 33% repeatability: 28% cut-off: 1.23 µg/kg false suspects for blanks: <0.1%	inter-laboratory	519/2014/EC	STC: 2 µg/kg reproducibility: 25% repeatability: 19% cut-off: 1.08 µg/kg false suspects for blanks: <0.1%	prEN 17279:2018 [17]

* Sum of repeatability + day-to-day variation + matrix-to-matrix variation.TC: training course intermediate precision: repeatability + within technician (sample preparation) variation.

**Table 3:** Method protocol comparison.

Mycotoxin/Matrix	Assay	Sample Size	Extraction	Additional Steps	Analysis	Calibration Curve	Results
DON/wheat	FPIA	25 g	100 mL PBS solution 2 min high speed blending	extract filtration through paper filter and glass microfiber filter	pipette: 830 µL PBS solution 80 µL antibody 120 µL filtered extract read the blank add 25 µL tracer result reading	provided by the TC organizers	evaluated trough an excel file*(input data: fluorescence polarization of the test sample)
	ELISA	5 g	100 mL water 3 min manual shaking	extract filtration through paper filter	pipette: 50 µL filtered extract 50 µL conjugate 50 µL antibody 250 µL wash buffer 100 µL substrate/chromogen 100 µL stop solution result reading	each technician made its own calibration	evaluated trough a software * (input data: absorbance of the calibration standards and the test sample)
	LFD	1 g	15 mL buffer * 3 min manual shaking		100 µL onto the test strip 5 min test strip development reading by smartphone	uploaded through bar code	provided by the smartphone app *
AFB_1_/maize	LFD	5 g	25 mL buffer * 2 min vortexing	extract filtration through paper filter	100 µL onto the test strip 5 min test strip development reading by optical reader	uploaded through a bar code	provided by the reader
DON/wheat AFB_1_/wheat	LC-HRMS	5 g	10 mL water + 10 mL acetonitrile 30 min shaking	5 g magnesium sulfate centrifugation	200 µL acetonitrile extract 20 µL ^13^C-IS solution 180 µL water inject into LC-HRMS	One point internal calibration (13C-IS addition)	evaluated trough an excel file * (input data: peak area of the mycotoxin in the test sample and peak area of the relevant 13C-IS)

* provided by the supplier (or by the training course organizers).
